# SUMOylation Blocks the Ubiquitin-Mediated Degradation of the Nephronophthisis Gene Product Glis2/NPHP7

**DOI:** 10.1371/journal.pone.0130275

**Published:** 2015-06-17

**Authors:** Haribaskar Ramachandran, Konstantin Herfurth, Rudolf Grosschedl, Tobias Schäfer, Gerd Walz

**Affiliations:** 1 Department of Medicine, Renal Division, University of Freiburg Medical Center, 79106 Freiburg, Germany; 2 Max-Planck-Institute of Immunobiology and Epigenetics, Stübeweg 51, D-79108 Freiburg, Germany; 3 BIOSS Center for Biological Signaling Studies, 79108 Freiburg, Germany; George Washington University, UNITED STATES

## Abstract

Glis2/NPHP7 is a transcriptional regulator mutated in type 7 nephronophthisis, an autosomal recessive ciliopathy associated with cystic and fibrotic kidney disease as well as characteristic extrarenal manifestations. While most ciliopathy-associated molecules are found in the cilium, Glis2/NPHP7 presumably localizes to the nucleus. However, the detection of endogenous Glis2/NPHP7 has remained unsuccessful, potentially due to its ubiquitylation-dependent rapid degradation. We report now that Glis2/NPHP7 is also SUMOylated, preferentially by PIAS4, which conjugates Glis2/NPHP7 to SUMO3. SUMOylation interferes with ubiquitylation and degradation of Glis2/NPHP7, suggesting that Glis2/NPHP7 protein levels are regulated by competing ubiquitylation/ SUMOylation. SUMOylation also alters the transcriptional activity of Glis2/NPHP7. While Glis2/NPHP7 activates the mouse insulin-2-promotor (mIns2), SUMOylated Glis2/NPHP7 lacks this property, and seems to act as a repressor. Taken together, our data reveal that Glis2/NPHP7 is extensively modified by post-translational modifications, suggesting that a tight control of this transcriptional regulator is required for normal development and tissue homeostasis.

## Introduction

Glis2/NPHP7 is one of three proteins that constitute the family of Glis-similar proteins (Glis1-3), a sub-family of Krüppel-like transcription factors characterized by five tandem Cys2/His2 zinc fingers (reviewed in [1]). Glis1-3 family members share a high degree of homology in their zinc finger region while show a very little sequence homology outside their zinc finger domain. (reviewed in [1]). While all three Glis proteins are expressed in the kidney, Glis2/NPHP7 mRNA is also detectable in several extra-renal tissues [2,3]. Consistent with their function as transcriptional regulators, Glis proteins localize predominantly to the nucleus, requiring the integrity of the zinc finger domains [4]. Glis2 contains a putative transactivation and repressor domain between amino acids 71 and 137 [3]. While Glis2 represses the Gli1-mediated activation of a reporter construct containing Glis-binding sequences, Glis2 activates the mouse Insulin-2 promoter [4], a property that Glis2 shares with Glis3 [5].

Positional cloning revealed that mutations of Glis2/NPHP7 cause type 7 nephronophthisis [6], an autosomal recessive condition associated with cystic kidney disease and several extra-renal manifestations, including retinitis pigmentosa and cerebellar abnormalities [7]. While nephronophthisis (NPH)^4^ is the most common cause of hereditary end-stage renal disease in children, only two families have been identified so far with Glis2/NPHP7 mutations [8]. Glis2/NPHP7-deficient mice develop glomerular cystic lesions in combination with severe renal fibrosis and atrophy [6]. Gene profiling experiments revealed that the lack of Glis2/NPHP7 is associated with an upregulation of genes associated with epithelial-to-mesenchymal transition (EMT), suggesting that Glis2/NPHP7 suppresses EMT to preserve an epithelial phenotype and to maintain normal kidney architecture [6]. Most gene products associated with nephronophthisis (NPH) localize to the primary cilium, a microtubular organelle present on epithelial cells. Hence, NPH together with related syndromes have been collectively termed ciliopathies, and Glis2/NPHP7, although predominantly present in the nucleus, has also been identified in the cilium [6]. Glis2/NPHP7 shares this localization with a second family member, Glis3. Glis3 deficiency in humans and mice is also associated with typical ciliopathy phenotypes, including cystic kidney disease [9–11], suggesting that both Glis2/NPHP7 and Glis3 are required for normal ciliary function and/or ciliary signaling. While the related Gli transcription factors require the cilium for proteolytic processing and activation (reviewed in [12,13]), it remains unknown whether Glis family members are regulated in a similar fashion.

Multiple interaction partners have been identified for Glis family members. The C-terminal binding protein 1 (CtBP1) interacts with Glis2, and appears to recruit HDAC3 to support the function of Glis2 as transcriptional repressor [14]. Glis2 also interacts with p120 catenin, promoting the import of p120 catenin into the nucleus [15]. Although the physiological consequences of this interaction remain unclear, Glis2 could participate in regulation of Rho GTPases, E-cadherin stability, Wnt signaling and EMT through binding partners of p120 catenin [1]. The E3 ubiquitin ligase scaffolding protein Cullin 3 recognizes the N-terminus of Glis3, and facilitates Glis3 polyubiquitination, while the Hedgehog regulator Suppressor of Fused (SuFu) interferes with the Cullin 3/Glis3 interaction, promoting the accumulation of Glis3 [5]. While SuFu also interacts with Glis2/NPHP7, this interaction does not seem to modify the stability and protein levels of Glis2/NPHP7.

One of the major problems to delineate the function of Glis2/NPHP7 is the inability to detect endogenous Glis2/NPHP7 protein by either immunofluorescence or Western blot analysis. Since Glis2/NPHP7 is ubiquitylated and targeted for proteasomal degradation [16], a short half-life and low protein levels may account for this problem. To further investigate the regulation of Glis2/NPHP7 proteins levels, we investigated whether Glis2/NPHP7 is modified by SUMOylation in addition to ubiquitylation. We report now that Glis2/NPHP7 interacts with the E3 SUMO ligase PIAS4, and is SUMOylated on conserved consensus sumoylation sites. SUMOylation inhibits Glis2/NPHP7 ubiquitylation, prolonging the half-life of this transcriptional repressor.

## Material and Methods

### Reagents and plasmids

MG132 (calbiochem), cycloheximide and N-Ethylmaleimide (Sigma-Aldrich) were used at concentrations as indicated. *In vitro* SUMOylation reaction was performed using a kit from Enzo life sciences. Full length human Glis2 (NM_032575) was synthesized by GeneArt (Life Technologies). Full length and truncated versions of Glis2 were created by PCR and standard cloning techniques. The cDNAs were fused to YFP (eYFP-C1 Clonetech), FLAG (PCDNA6, Invitrogen) and V5 (PCDNA6, Invitrogen). Glis2 with mutations in SUMO consensus sequences were generated by quick change PCR using Glis2 wild type as template. Luciferase reporter with mIns2 promoter was kindly provided by Dr.Fererri Kevin. Ubiquitin was cloned into a HA tagged pMT123 plasmid using *NotI* and *ECORI* restriction sites. GST.Glis2 was generated by cloning full length Glis2 into GST-pGEX4T1 using *Mlu* and *NotI* sites. Pre.SUMO-1, 2 and 3 were cloned into PCDNA4/TO/N-MRGS-6xHis vector. Full length PIAS4 and PIAS1 in PCDNA6 with different tags was generated using CDNA clones from Imagenes. PIAS4 lacking E3 ligase activity was generated by quick change PCR using PIAS4 wild type as template. Antibodies used in this study included anti-FLAG M2 (1:3000, Sigma-Aldrich), anti-V5 (1:6000, Serotec) anti-GFP(1:1000, MBL) anti-β-actin (1:1000, Sigma-Aldrich) anti-HA 12CA5 (1:1000, Roche Applied Sciences), anti-αtubulin (1:2000, Sigma-Aldrich) anti-GST (1:1000-Amersham), anti-acetylated tubulin and anti-SUMO-3 (1:1000, Abcam). Secondary horseradish peroxidase (HRP)-coupled antibodies against rabbit and mouse IgG were from Dako and GE Healthcare and were used at dilution of 1:10,000. Cy3 and Cy5 conjugated antibodies (1:500) were from Jackson ImmunoResearch

### Cell culture and transfections

Dulbecco’s Modified Eagle Medium (DMEM) supplemented with 10% Fetal Bovine Serum (FBS) (Biochrome) was used to culture HEK 293T cells. DMEM-F12 supplemented with 10% FBS was used to culture mIMCD3 cells. The cells were transfected with using Calcium phosphate or TransPEI transfection method (Eurogentec, Cologne, Germany). Stably transfected mIMCD3 cells were generated using retroviral transduction by three consecutive transduction cycles and the transduced cells were selected using puromycin.

### Co-immunoprecipitation and Western blotting

After 24hrs of transfection, cells were washed with PBS and lysed using lysis buffer (20 mM Tris, pH 7.5, 1% Triton X-100, 50 mM NaCl, 50 mM NaF, 15 mM Na_4_P_2_O_7_, 0.1 mM EDTA) supplemented with protease inhibitor cocktail complete (Roche). After centrifugation (15000g 15min 4°C) followed by ultracentrifugation (100,000g 30min 4°C), the lysates were incubated with 30μl of FLAG M2 sepharose beads (Sigma) for about 2hrs at 4°C in an overhead shaker. After incubation, the beads were washed with lysis buffer and the proteins were eluted in 2x-lämmeli buffer supplemented with β-mercaptoethanol or DTT. The bound proteins in elutes were further resolved by SDS-PAGE and the interaction was checked by Western blotting using respective antibodies.

### Wheat germ cell free expression system for *in vitro* interaction studies

FLAG-tagged Glis2, V5-tagged PIAS4 and V5-tagged CD2AP were produced using RTS 100 Wheat Germ CECF Kit (5Prime GmbH, Hamburg, Germany) according to the manufacturer’s instructions. Co-immunoprecipitation of V5 tagged proteins was perfromed as described above, using V5 agarose beads; the protein expression and interaction was tested by Western blotting.

### SUMOylation assay

HEK 293T cells were transiently transfected with the plasmids to be investigated and the SUMO machinery (His.SUMO-3, and YFP.PIAS4). After 36hrs of transfection, the cells were washed with PBS and subjected to lysis with SUMO lysis buffer (150mM-NaCl, 25-mMTris-HCl (pH 7.8), 0.1% Nonidet-P40, 1 mM EDTA, 0.2% SDS, 10mM N-Ethylmaleimide) on ice for 30min. The highly viscous lysate due to the release of chromatin was further clarified by sonication for 5 seconds (Setting 3, Branson Sonifier 450) and centrifuged at 13000 rpm for 30 min. After centrifugation whole cell lysates were incubated at 37°C with 2X Lämmeli buffer containing DTT. Proteins were resolved in a 10% SDS-PAGE gel and stained using anti-V5 antibody to detect the presence of SUMOylated forms. For co-immunoprecipitation, the transfected cells were lysed as above and Ni^2+^-NTA agarose (Qiagen) was used to precipitate His tagged SUMO proteins. In vitro SUMOylation assay was performed with 200 nM of recombinant GST.Glis2 using a commercially available kit (Enzo life sciences) according to manufacturer instructions.

### Ubiquitylation assays

HEK 293T cells were transfected with the plasmids to be investigated along with HA tagged ubiquitin construct. After 24hrs of transfection, the cells were washed with PBS and subjected to lysis using RIPA buffer (1% Triton X-100, 0.5% sodium deoxycholate, 0.1% SDS, 150 mM NaCl, 50 mM NaF, 2 mM EDTA, 13.7 mM Na_2_HPO_4_, 6.3 mM NaH_2_PO_4_). The lysates were clarified by ultracentrifugation and incubated with FLAG-M2 beads at 4°C for 2hrs. After washing four times with lysis buffer, bound proteins were further resolved by SDS-PAGE gel and stained with HA antibody (Roche) to detect ubiquitylated proteins.

### Immunofluorescence

HEK 293T cells were cultured on six well plates containing coverslips pre-treated with polylysine in order to ensure the tight attachment of the cells. After 24hrs of transfection, cells were fixed with 4% PFA for 15 minutes, and permeablized with 0.25–0.5% Triton-X containing PBS. The cells were then blocked with (2% horse serum+0.5% Tween20) and incubated with primary antibody for 1hr. After washing off the primary antibody, secondary antibody was added and further incubated for 30-45min. After washing off the excess secondary antibody, nuclear staining was performed using DAPI. After several washing steps, coverslips were mounted on a clear microscopic slide using prolong gold anti-fade reagent (Invitrogen). mIMCD3 cells were cultured on six well plates for about 5 days and stained with acetylated tubulin as described above. Images were captured with LSM 510 confocal microscope (Zeiss, Germany) and ApoTome microscope (Zeiss, Germany).

### Luciferase assay

HEK 293T cells were split into 12 wells and transiently transfected with the plasmids to be investigated in triplicates along with the luciferase reporter construct and a β-galactosidase expression vector. After 24hrs of transfection, the cells were lysed with Tropix lysis buffer (Applied Biosystems) for 15min at room temperature. Lysates were centrifuged at 15000g for 5min. Supernatant was transferred to a 96 well plate and luciferase activity was measured and normalised to β-galactosidase activity to correct for transfection efficiency.

### Statistical analysis and quantification

For Western blotting, the blots were scanned and signals were quantified using LabImage 1D- (Intas Science Imaging) and normalised to actin controls. For MG132 experiments, data were analysed using Student’s t-test (two tailed, unpaired). Error bars represent standard deviation. For luciferase assays, data from three independent experiments were analysed using Ordinary one way ANOVA followed by Turkey’s multiple comparison test.

## Results

### Glis2 interacts with PIAS4 and SUMO-3

**Fig 1:**
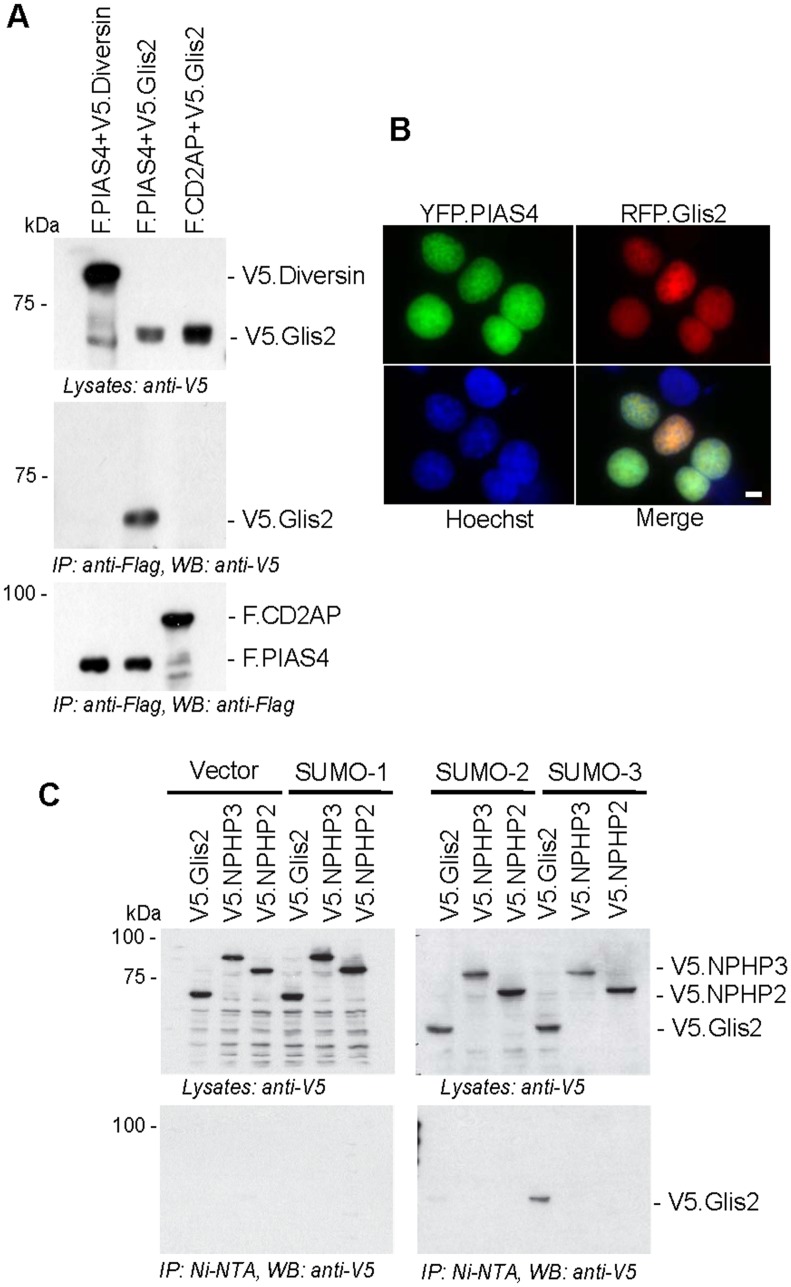
Glis2 interacts with PIAS4 and SUMO-3. (A) FLAG-tagged PIAS4 (F.PIAS4) and V5-tagged Glis2 (V5.Glis2) were transiently co-expressed in HEK 293T cells together with F.CD2AP and V5.Diversin as controls. Precipitation of F.PIAS4 immobilized only V5.Glis2 but not V5.Diversin, while V5.Glis2 was not detectable in F.CD2AP precipitates. (B) Immunofluorescence analysis of HEK 293T cells expressing YFP.PIAS4 (green) and RFP.Glis2 (red) showed nuclear co-localisation of both proteins. Nuclei were stained with Hoechst blue. (C) His-tagged SUMO proteins (SUMO-1, SUMO-2, and 3) were transiently co-transfected with V5.Glis2, V5.NPHP3 and V5.NPHP2 in HEK 293T cells. After 36 hours of transfection, SUMO proteins were precipitated using Ni-NTA beads. Glis2 and other nephronophthisis gene products (NPHP) were detected using an anti-V5 antibody.

We recently identified TRIM32 as a binding partner of Glis2 [16]. Since TRIM32 interacts with the E3 SUMO ligase PIAS4/PIASγ [17] and SUMOylation often targets functionally or physically connected proteins [18], we examined whether Glis2 directly or indirectly interacts with PIAS4 and SUMO machinery. FLAG-tagged PIAS4 and V5-tagged Glis2 were transiently co-expressed in HEK 293T cells. Flag-tagged CD2AP, a signalling scaffold protein implicated in variety of physiological processes and V5-tagged Diversin, an ankyrin-repeat protein involved in planar cell polarity signalling, were used as negative controls. F.PIAS4 interacted with V5.Glis2, but not with the control proteins V5.Diversin and F.CD2AP (Fig 1A). The direct physical interaction between PIAS4 and Glis2 was further confirmed by co-immunoprecipitation, using proteins produced by cell-free wheat germ system (S1A Fig).Co-localisation of fluorescently tagged Glis2 and PIAS4 in the nucleus further supported a functional interaction between these two proteins (Fig 1B). We next examined whether Glis2 interacts with SUMO family members. Co-expression with SUMO proteins in HEK 293T cells and pull-down under non-denaturing conditions revealed that Glis2, but none of the other control nephrocystins interacted with SUMO-3 (Fig 1C).

**Fig 2:**
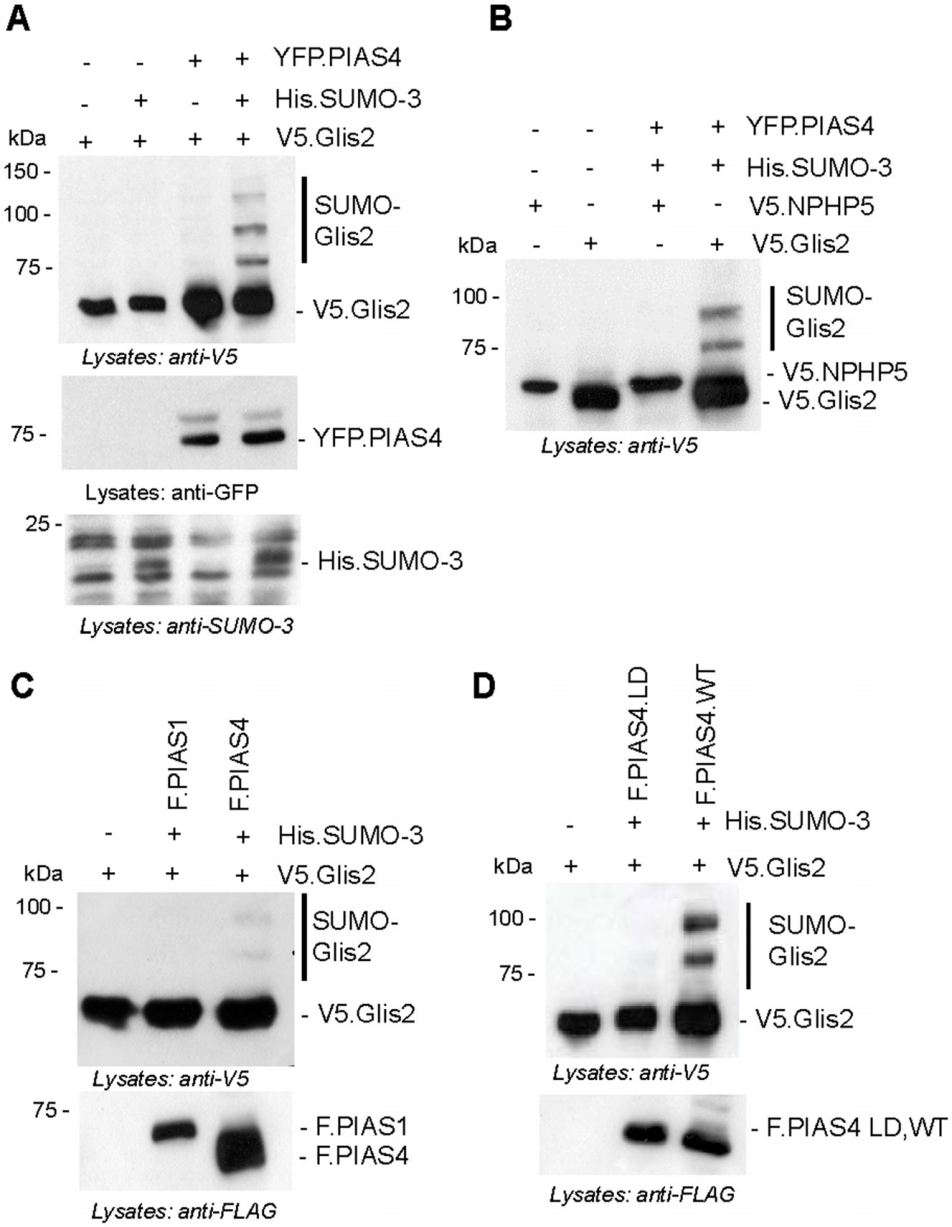
Glis2 is SUMOylated by PIAS4/SUMO-3. (A) HEK 293T cells were transfected with the plasmids as indicated. After 36 hours of transfection, whole cell lysates were prepared under denaturing conditions, and resolved on SDS-PAGE. Following Western blotting, Glis2 was detected using an anti-V5 antibody, YFP.PIAS4 using an anti-GFP antibody, and SUMO-3 using an anti-SUMO-3 antibody. SUMOylated Glis2 species are marked as SUMO-Glis2. (B) *In vivo* SUMOylation assay was performed to compare the SUMOylation of Glis2 and NPHP5 in the presence of SUMO-3 and PIAS4. Only SUMOylated Glis2 species, but no SUMOylated NPHP5 was detectable. (C) Co-transfection of FLAG-tagged PIAS4 (F.PIAS4) but not F.PIAS1 promoted the SUMOylation of Glis2 in the presence of SUMO-3. (D) V5.Glis2 was co-transfected with F.PIAS4 wild type (F.PIAS4.WT) and PIAS4, lacking E3 SUMO ligase activity (F.PIAS4.LD) together with His.SUMO-3. SUMOylated Glis2 was detected only with the wild type but not with the E3 ligase-deficient mutant of PIAS4.

*In vivo* SUMOylation assays, involving cell lysis under denaturing conditions in the presence of SUMO protease inhibitor N-Ethylmalemide(Sigma) demonstrated the presence of SUMOylated Glis2 species in the presence of SUMO-3 and PIAS4. SUMOylated Glis2 appeared as slow-migrating bands above V5-tagged Glis2 (Fig 2A). Faint bands occurred in the presence of PIAS4 without co-expression of His.SUMO-3, but clearly detectable higher molecular weight Glis2 species were only detectable, if V5-tagged Glis2 was co-expressed with both SUMO-3 and PIAS4. These slow-migrating bands were detected only for Glis2, but not for NPHP5, another nephronophthsis family member of comparable molecular weight (Fig 2B). PIAS4 but not PIAS1 served as an E3 SUMO ligase for Glis2 (Fig 2C). SUMOylation of Glis2 was abolished in the presence of PIAS4 ligase-dead (LD) mutant (Fig 2D). These observations suggest that Glis2 is preferentially conjugated to SUMO-3 in the presence of the E3 SUMO ligase PIAS4.

### *In vitro* Glis2 SUMOylation

**Fig 3:**
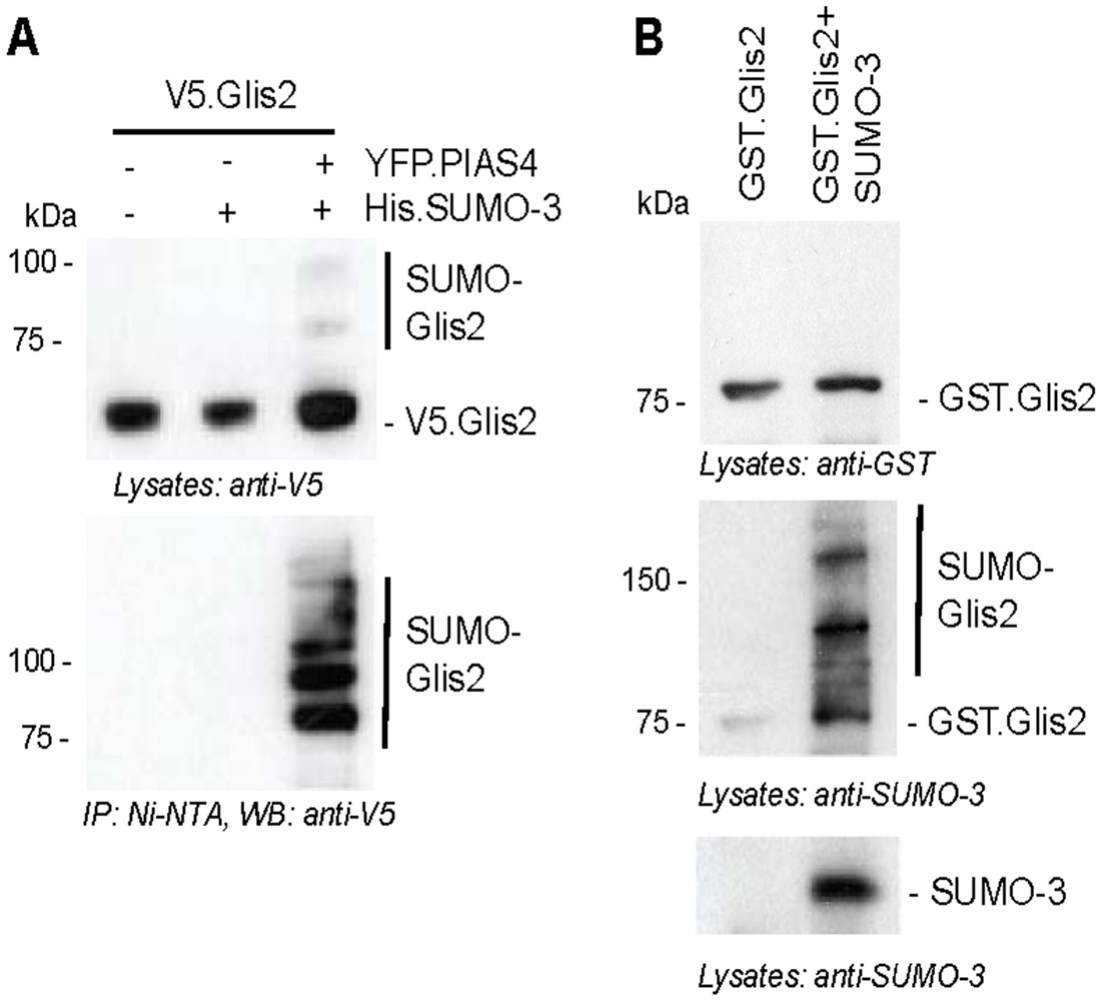
Glis2 is SUMOylated *in vitro*. (A) HEK 293T cells were transiently transfected with the indicated plasmids. Whole cell extracts were prepared under denaturing conditions and incubated with Ni-NTA beads. Purified proteins were resolved on SDS-PAGE. Following Western blotting, Glis2 was detected using an anti-V5 antibody. (B) *In vitro* SUMOylation of recombinant GST.Glis2 was analyzed using a commercially available SUMOylation kit. Glis2 was detected using an anti-GST antibody, and SUMOylated Glis2 was detected using an anti-SUMO-3 antibody.

Co-expression and pull-down of His.SUMO-3 in the presence of PIAS4 in HEK 293T cells using nickel beads immobilized only SUMO-3 containing, V5-tagged Glis2, confirming the conjugation of SUMO-3 to Glis2 *in vivo* (Fig 3A). To further support the conjugation of Glis2 to SUMO-3, we performed *in vitro* SUMOylation, using recombinant GST.Glis2 in combination with a commercially available SUMOylation kit (Biomol). Addition of SUMO-3 to the reaction mix resulted in SUMOylated GST-tagged Glis2, confirming the SUMOylation of Glis2 in the presence of SUMO-3 (Fig 3B).

### Glis2 is SUMOylated at multiple sites by SUMO-3

**Fig 4:**
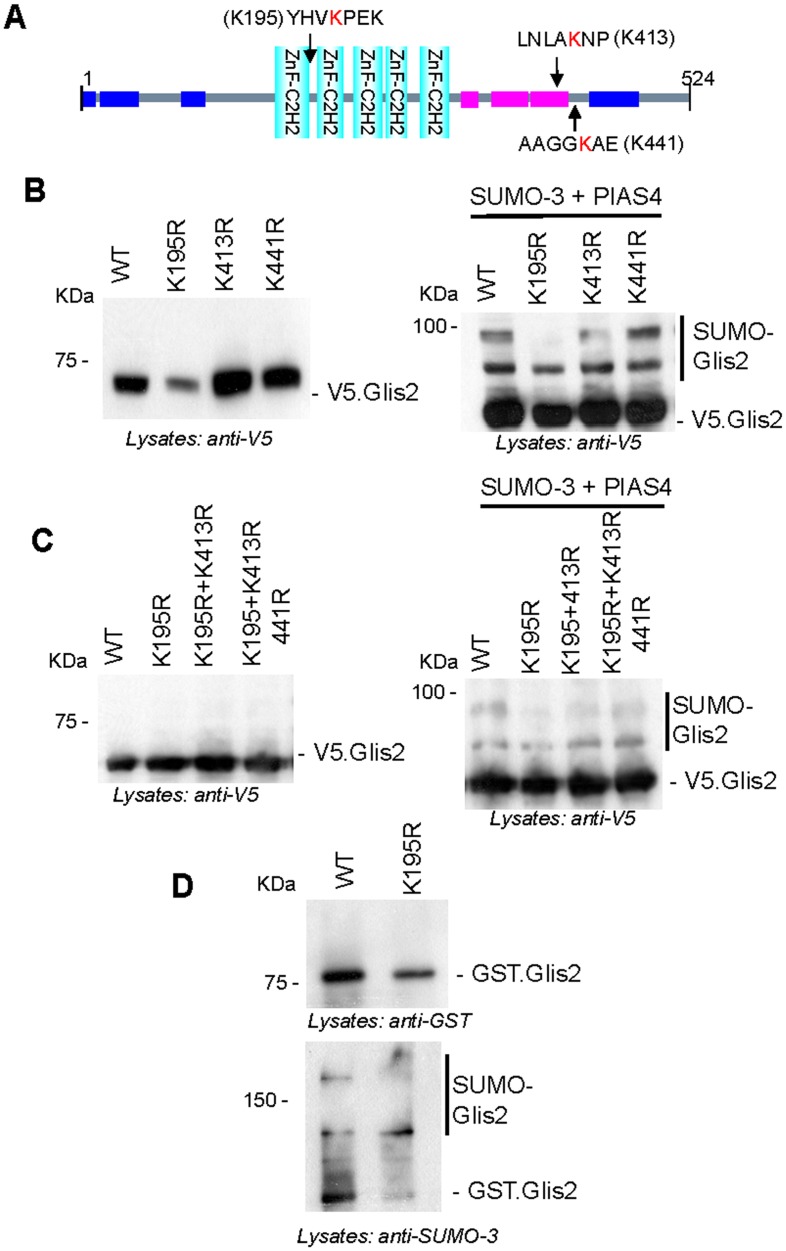
Lysine 195 contributes to Glis2 SUMOylation. (A) Consensus SUMOylation sites of Glis2 predicted by SUMOplot. (B) HEK 293T cells were transfected with either Glis2 wild type (WT), or Glis2 mutants, replacing the predicted target lysines with arginine (K195R, K413R and K441R) together with SUMO-3 and PIAS4. After 36 hours of transfection, whole cell extracts were prepared under denaturing conditions, and Glis2 was detected using a V5 antibody. (C) *In vivo* SUMOylation assay demonstrated that the combined mutagenesis of different target lysines did not completely abolish Glis2 SUMOylation, suggesting additional SUMOylation sites not predicted by SUMOplot. (D) *In vitro* SUMOylation assay using GST.Glis2 Wild type (WT) and GST.Glis2.K195R confirmed that K195 of Glis2 is targeted by SUMOylation.

The presence of two distinct higher molecular forms in the in vivo SUMOylation assays indicated that Glis2 might be SUMOylated on more than one site, or subjected to an additional posttranslational modification. We recently described that Glis2 is polyubiquitylated (16). Since polySUMOylation of Glis2 is also possible, we used SUMOplot^T^ (Abgent-http://www.abgent.com/sumoplot) to predict potential consensus SUMOylation sites in Glis2. Glis2 contains three predicted ΨKx(D/E) SUMOylation consensus sites (SUMOplot) at K195 (Score: 0.93), K413 (Score: 0.69) and K441 (Score: 0.63) (Fig 4A). Mutation of these three sites showed that the K195R mutation, but neither of the other two lysine substitutions influenced the detection of SUMOylated Glis2 species (Fig 4B). However, even the triple mutant (K195R/K413R/K441R) did not prevent SUMOylation, suggesting that Glis2 can be SUMOylated on additional sites not predicted by SUMOplot (Fig 4C). Although the *in vitro* SUMOylation assay confirmed that K195 represents a SUMOlyation site of Glis2 (Fig 4D), our mutational analysis does not provide conclusive evidence for the functional importance of this site.

### SUMOylation interferes with Glis2 ubiquitylation

**Fig 5:**
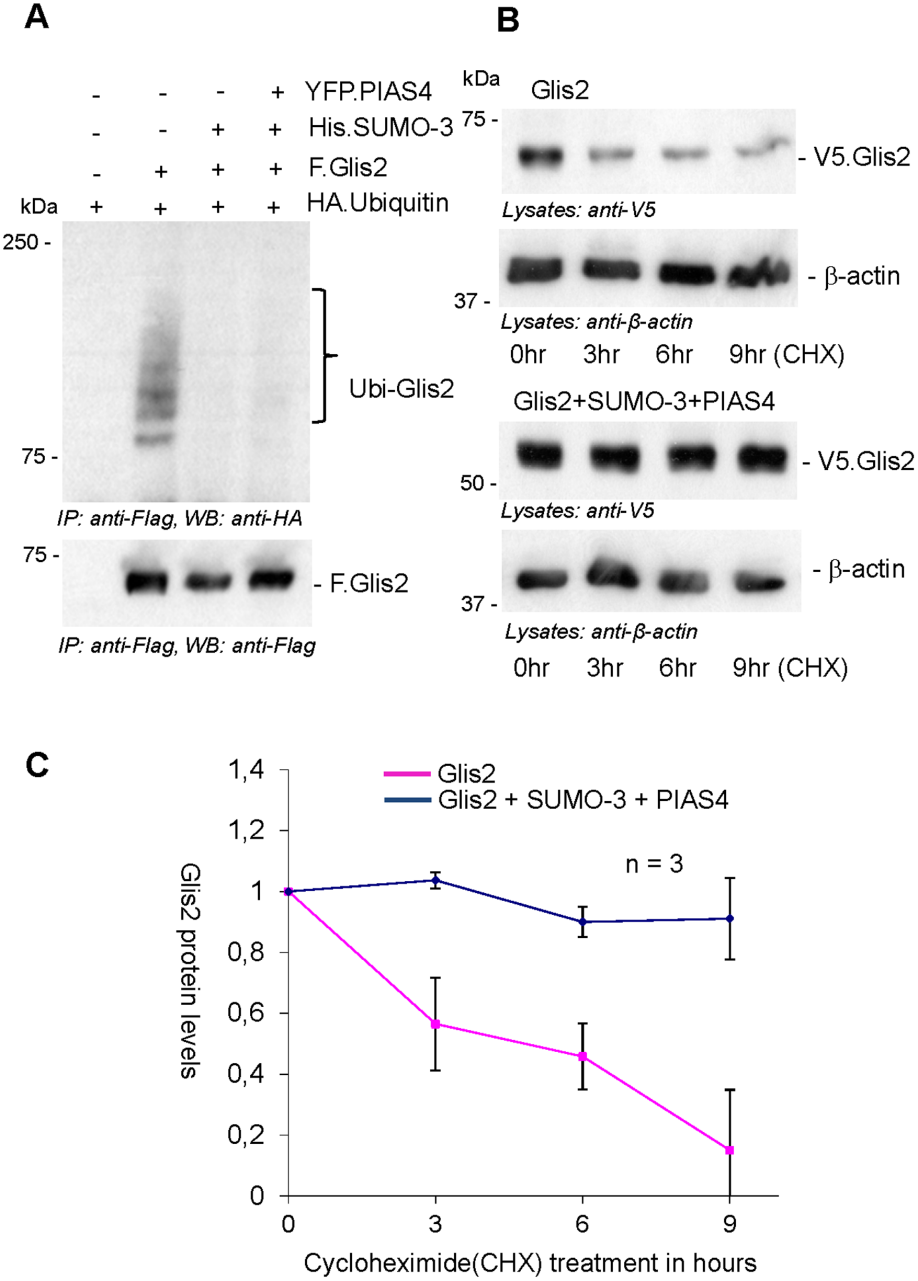
SUMO-3 blocks the ubiquitylation of Glis2. (A) HEK 293T cells were transfected with the plasmids as indicated. After 24 hours of transfection, F.Glis2 was precipitated using FLAG-M2 beads; Glis2 ubiquitin species were detected using anti-HA antibody (Ubi-Glis2, top). (B) HEK 293T cells transfected with Glis2 alone and with SUMO-3 and PIAS4 were treated with cycloheximide (30 μg) to prevent protein synthesis. After 9 hours of treatment, protein turnover of Glis2 was analyzed by Western blotting using a V5 antibody. Anti-β-actin staining confirmed equal protein loading. (C) The graph demonstrates the fraction of Glis2 remaining after 3 to 9 hours of cycloheximide treatment in the presence or absence of SUMO-3/PIAS4.

SUMO can antagonize ubiquitylation by competing for the lysine residues to which ubiquitin binds, and prevent ubiquitin-dependent proteasomal degradation. For example, SUMO-1 has been shown to control the stability of IkBα, an inhibitor of NF-κB by blocking lysine 21 [19]; in addition, there is evidence for a functional cooperation between these two pathways. The E3 ubiquitin ligase RNF4 recognizes SUMO polymers attached to PML, and recruits the ubiquitin proteasome to degrade PML in response to arsenic treatment [20]. We examined whether SUMOylation primes Glis2 for ubiquitin-dependent degradation. Ubiquitylation of Glis2 was almost completely abrogated when SUMO-3 was co-expressed with Glis2 (Fig 5A), suggesting that SUMOylation inhibits Glis2 ubiquitylation and prevents ubiquitin-dependent recognition and degradation of Glis2 by the proteasome. We performed cycloheximide chase experiments to examine the half-life of Glis2 in the presence of the SUMOylation machinery. Co-expression of SUMO-3 and PIAS4 resulted in a prolongation of the half-life of Glis2 (Fig 5B and 5C). This observation suggests that SUMOylation prevents subsequent ubiquitylation and degradation of Glis2.

### The K195R mutation reduces Glis2 stability

**Fig 6:**
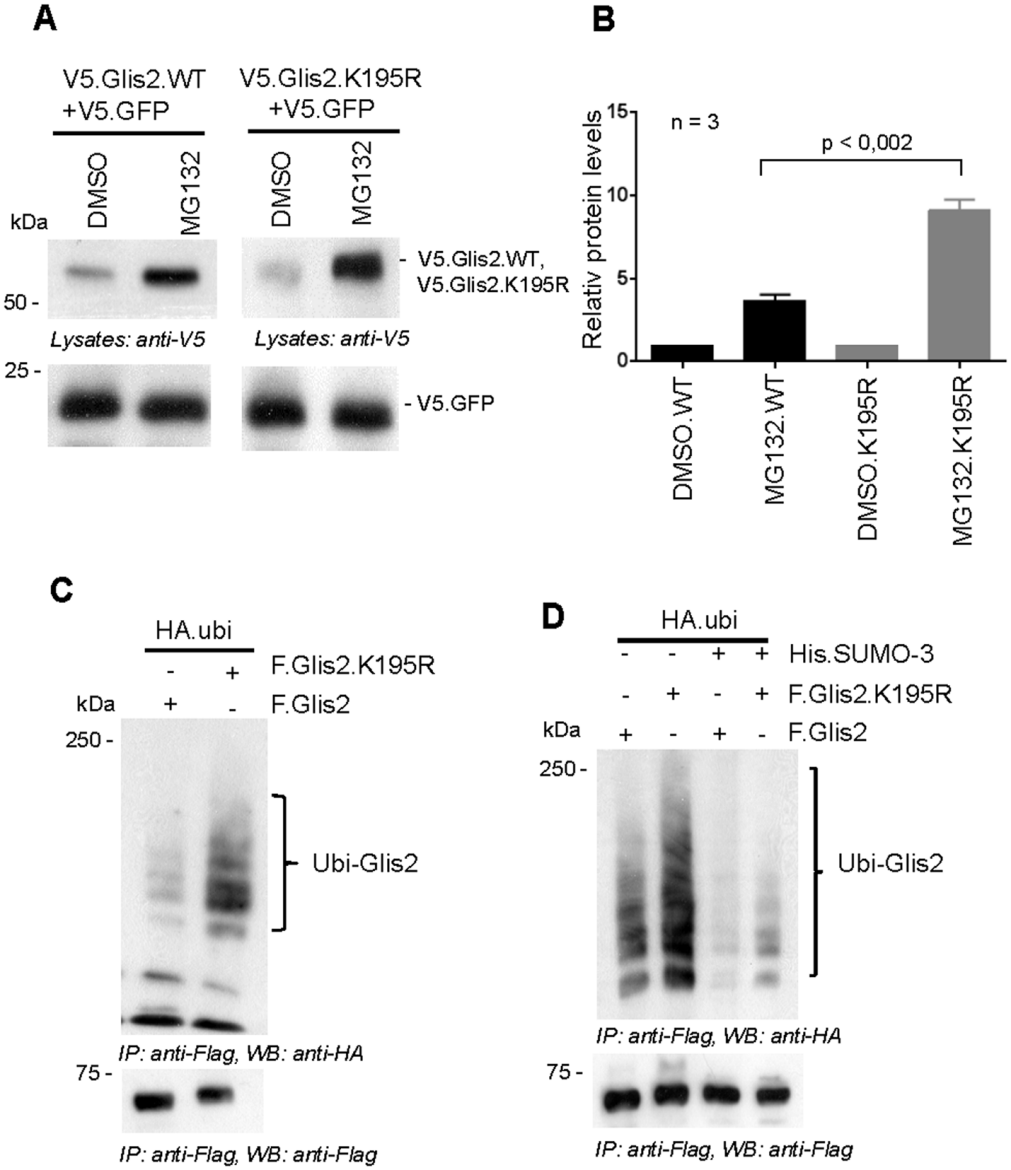
Lysine 195 is required for Glis2 stability. (A) HEK 293T cells, transfected with Glis2 wild type (WT) or Glis2.K195R along with V5-tagged GFP, were treated with MG132 (12 μM) for 2 hours to inhibit proteasomal degradation. DMSO was used as a vehicle control. The cell lysates were resolved on SDS-PAGE, and the protein levels of Glis2 were detected using anti-V5 antibody. (B) Bars represent the quantification of Glis2.WT and Glis2.K195R protein levels normalised to V5-tagged GFP levels from three independent experiments (n = 3). (C) *In vivo* ubiquitylation assays demonstrate the increased ubiquitylation of F.Glis2.K195R in comparison to Glis2 wild type (WT). (D) *In vivo* ubiquitylation assays were performed with the plasmids as indicated to compare the SUMO-3 mediated inhibition of ubiquitylation of F.Glis2 wild type (WT) and F.Glis2.K195R.

Protein levels of Glis2.K195R were consistently reduced when compared to wild-type Glis2, suggesting that the Glis2.K195R mutant is less stable than wild-type Glis2. Glis2.K195R protein levels were more strongly increased by the proteasome inhibitor MG132, supporting the role of SUMOylation in Glis2 stability (Fig 6A and 6B). To avoid differences in transfection efficiency, Glis2.WT and Glis2.K195R protein levels were normalized to co-transfected V5-tagged GFP. Furthermore, ubiquitylation of the Glis2 K195R mutant was increased in comparison to wild-type Glis2 (Fig 6C). Over-expression of SUMO-3 suppressed ubiquitylation of both wild-type and mutant Glis2 K195R (Fig 6D). This observation confirms that K195 represents a major Glis2 SUMOylation site, but the findings also suggest that there are additional functional SUMOylation sites.

### SUMOylation affects Glis2 function

**Fig 7:**
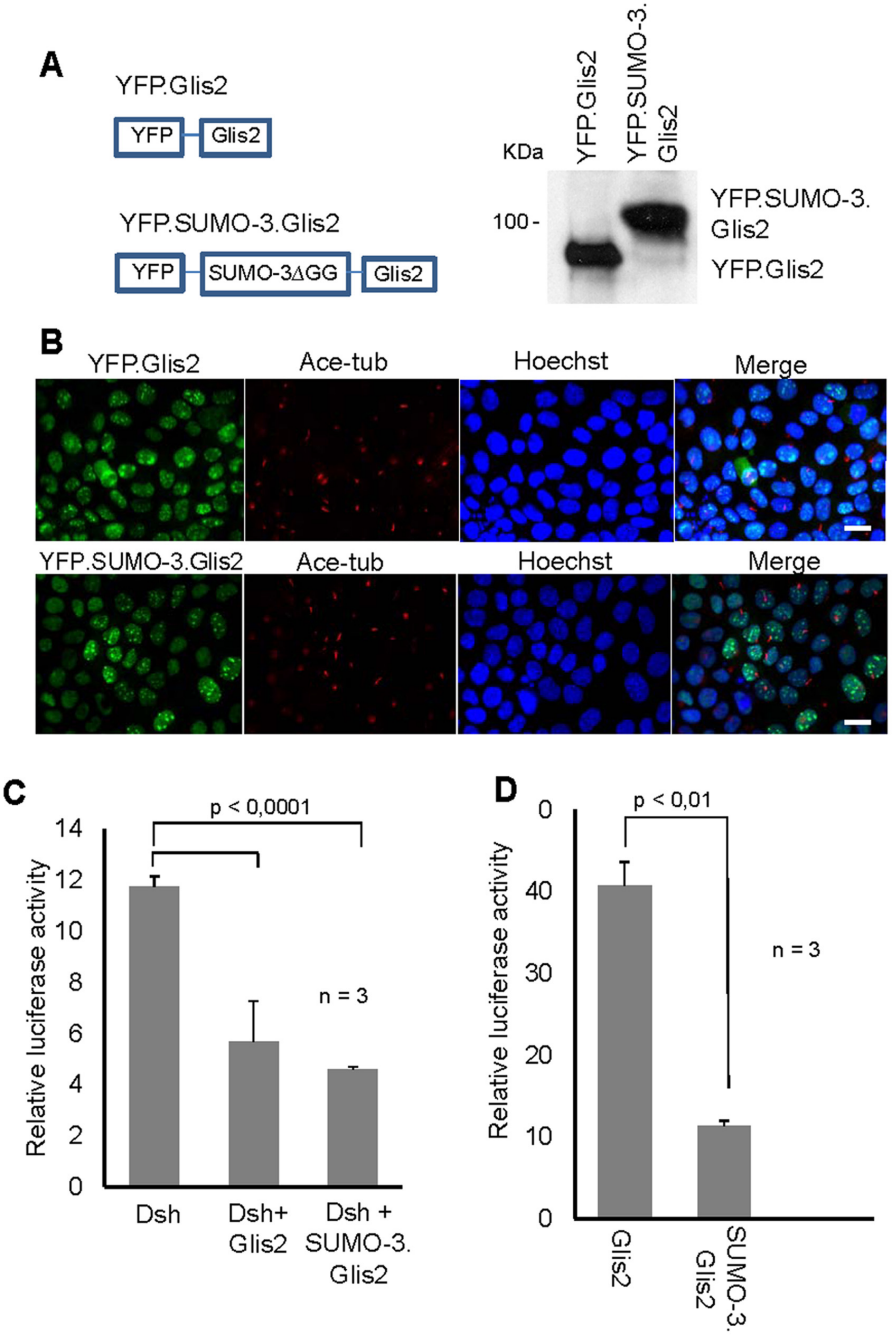
SUMO-3 fusion to Glis2 modulates the transcriptional activity of Glis2. (A) SUMO-3 was N-terminally fused to Glis2 to generate a constitutively SUMOylated Glis2. The di-glycine motif of SUMO-3 recognized by de-SUMOylating enzymes was also deleted to maintain the SUMOylation of Glis2. mIMCD3 cells stably transfected with YFP.Glis2 and YFP.SUMO-3.Glis2 were generated, and the expression of the respective proteins was confirmed by Western blotting using an anti-GFP antibody. (B) Co-localisation studies of YFP.Glis2 and YFP.SUMO-3.Glis2 with anti-acetylated tubulin (to mark cilium) in mIMCD3 cells. Glis2 was detected using an anti-GFP antibody, and Hoechst stain to label the nuclei. (C) HEK 293T cells were transfected with indicated plasmids to activate TCF/LEF dependent gene expression (TOPflash assay). Three independent experiments (n = 3) were performed in triplicates, and the values were normalised to β-gal levels. (D) Activation of mIns2 promoter by Glis2 and SUMO3-Glis2 was quantified from three independent experiments in HEK 293T cells.

**Fig 8:**
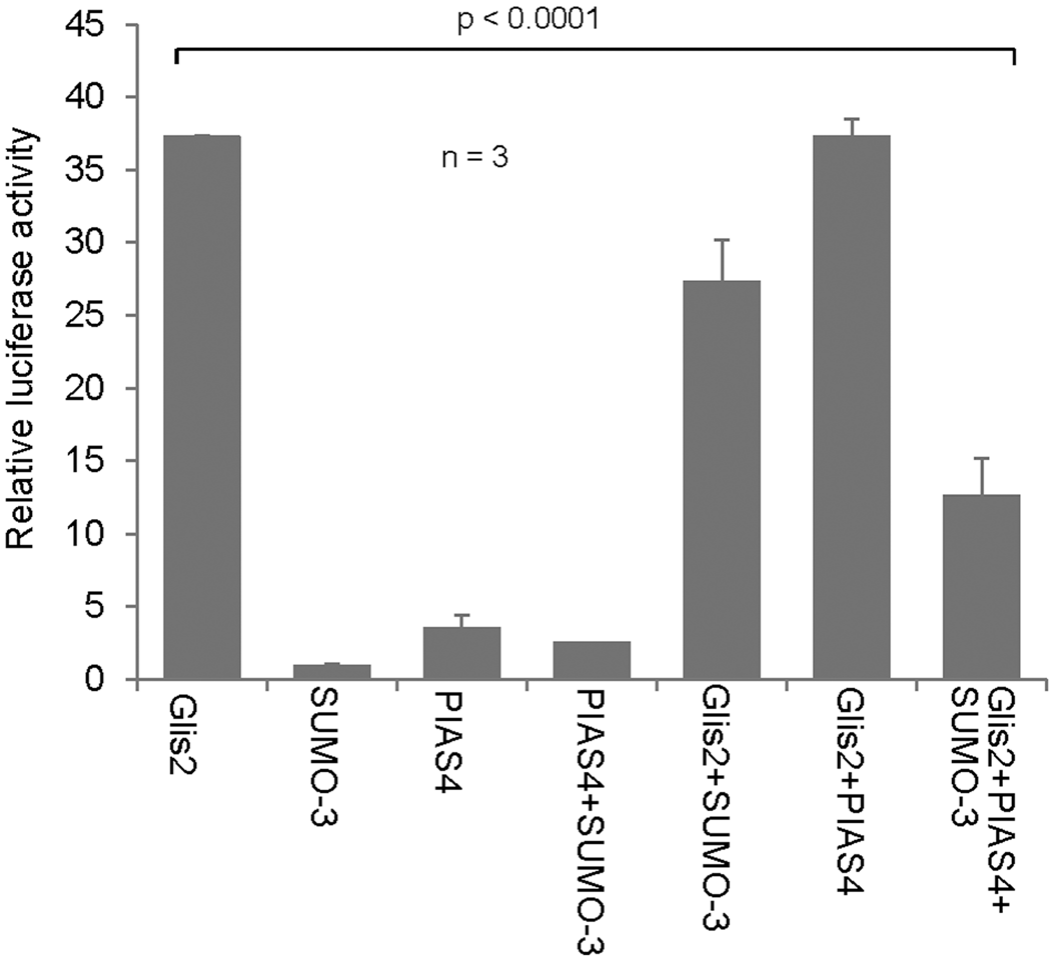
SUMO-3 and PIAS4 reduces the transcriptional activator function of Glis2. Co-transfection of PIAS4 and SUMO-3 reduced the transcriptional activator function of Glis2. Three independent experiments were performed with triplicates each time.

SUMOylation has recently been implicated as a ciliary targeting mechanism [21]. To test whether SUMOylation targets Glis2 to the cilium, we used mIMCD3, a mouse inner medullary collecting duct cell line that forms primary cilia. Since SUMO conjugates are short-lived, we generated a SUMO-3.Glis2 fusion protein (Fig 7A), and expressed both YFP-tagged Glis2 and SUMO-3.Glis2 in mIMCD3 lysates. Western blot analysis of these mIMCD3 lysates demonstrated that both full-length fusion proteins were expressed (Fig 7A). However, neither Glis2 nor SUMO-3 fused to Glis2 localized to the cilium labelled for acetylated tubulin (Fig 7B). Further examination of the SUMO.Glis2 fusion protein, exhibited no differences in ubiquitiylation as well as in stability in comparison to Glis2, using *in vivo* ubiquitylation and cycloheximide chase assays (S1B–S1D Fig). We next examined whether Glis2 SUMOylation alters the function of this transcriptional repressor. Glis2 suppresses the TCF/LEF-dependent gene transcription triggered by Dishevelled. Surprisingly, SUMO-3.Glis2 had did not alter the ability of Glis2 to suppress the Dishevelled-mediated TOPflash activity (Fig 7C). Glis2 not only functions as transcriptional repressor but has recently been shown to activate the murine insulin-2 promoter (mIns2) [4]. We noted that SUMOylation curtailed the ability of Glis2 to activate the mIns2 promoter by either the SUMO-3.Glis2 fusion protein or co-expression of SUMO-3 and PIAS4 (Figs 7D and 8). These findings suggest that SUMOylation does not interfere with the transcriptional repression by Glis2, but may influence its properties as transcriptional activator.

## Discussion

Mutations of Glis2/NPHP7 are a rare cause for nephronophthisis (NPH), an autosomal recessive disease associated with end-stage renal disease and variable extrarenal manifestations; so far, only two families with Glis2/NPHP7 mutations and typical NPH manifestations have been identified [8]. In addition, depletion of Glis2/NPHP7 causes renal cysts and fibrosis in mice [6] and typical ciliopathy phenotypes in zebrafish [22], supporting the involvement of Glis2/NPHP7 in NPH. However, Glis2/NPHP7 differs from other NPH family members. While most NPH proteins are associated with the cilium/centrosome complex, and function as adaptor proteins that assemble large protein networks [23], Glis2/NPHP7 represents a transcriptional repressor residing in the nucleus [4]. We have previous demonstrated that Glis2/NPHP7 interacts with TRIM32/BBS11 [16]. Since TRIM32/BBS11 also interacts with PIAS4/Piasγ [17], we speculated that TRIM32/BBS11 might recruit this E3 SUMO ligase to facilitate SUMOylation of Glis2/NPHP7. SUMOylation is a widely recognized post-translational modification characterized by attachment of ubiquitin-like molecules to the ε-amino group of lysine residues. Although cytosolic proteins can be SUMOylated, SUMO substrates are primarily nuclear proteins and transcription factors (reviewed in [24]). SUMOylation can interfere with other post-translational modifications such as acetylation or ubiquitylation, block or enhance interaction with other proteins, or trigger intra-molecular conformational changes to activate enzymatic activities. Our results reveal that Glis2/NPHP7 can be both ubiquitylated and SUMOylated. SUMOylation is mediated by a series of proteins similar to ubiquitylation. However, in contrast to ubiquitylation, Ubc9 is the only E2 that mediates SUMOylation of canonical ΨKx(D/E) sites without the presence of E3 ligases. Substrate specificity is typically mediated by E3 SUMO ligases, and consistent with this role of E3 SUMO ligases, we found that PIAS4 preferentially targets Glis2/BBS11. While we identified K195 as one of the three predicted motifs modified by SUMOylation, canonical sites are often directly SUMOylated by Ubc9; in addition, PIAS E3 SUMO ligases can also SUMOylate substrates on non-consensus sequences [24].

We have previously reported that recruitment of the RING-type ubiquitin ligase TRIM32 facilitates the accumulation of ubiquitylated Glis2/NPHP7 species. Although it remains unknown whether the Glis2/TRIM32 interaction requires prior Glis2/NPHP7 SUMOylation, SUMOylation of Glis2/NPHP7 does not seem to trigger SUMO-dependent recruitment of ubiquitin E3 ligases to target Glis2/NPHP7 for ubiquitylation on pre-existing SUMO chains and subsequent degradation. In fact, we observed that co-expression of SUMO-3/PIAS4 and SUMOylation dramatically reduced ubiquitylation, and prolonged the half-life of Glis2/NPHP7. Thus, our observations suggest that SUMOylation competes with ubiquitylation to prevent degradation of Glis2/NPHP7.

SUMOylation occurs at specific intracellular localizations. For example, the RANBP2 SUMOylation complex localizes to nuclear pores, where it appears to SUMOylate proteins during their nuclear import [25]. Several nuclear import mechanisms including SUMOylation have recently been implicated in recruitment of cargo molecules to the ciliary compartment [26]. To mimic constitutive SUMOylation [27], we generated a SUMO-Glis2/NPHP7 fusion protein, and tagged the construct with YFP. Western blot analysis confirmed that the full-length protein is expressed in virally transduced IMCD3 cells, which form cilia upon confluence. However, despite detection of the SUMO-Glis2/NPHP7 fusion in the nucleus, there was no detectable protein in the cilium, suggesting that SUMOylation does not appear to target Glis2/NPHP7 to the cilium. Since neither stability nor ubiquitylation of this fusion protein was altered, these results indicate that the presence of free SUMO-3 in the presence of PIAS4 is necessary to inhibit the ubiquitylation of Glis2.

Glis2/NPHP7 not only acts as a transcriptional repressor, but was recently shown to also activate the murine insulin-2 promoter [4]. Interestingly, a phospho-mimetic S245D mutation of Glis2/NPHP7 reduced its ability to activate the promoter, suggesting that the transcription-stimulatory activity of Glis2/NPHP7 is regulated by post-translational modifications. Consistent with this observation, we found that SUMOylation reduced the transcriptional activity of Glis2/NPHP7, while it had no apparent effect on the transcriptional repression of Glis2/NPHP7.

Glis2/NPHP7 is mutated in nephronophthisis, a hereditary condition associated with cystic kidney disease, renal fibrosis, and various extrarenal manifestations. Glis2/NPHP7 differs from the other NPHP family members by its predominant nuclear localization, and function as the transcriptional repressor/activator. Endogenous Glis2/NPHP7 protein seems to occur only at very low levels; although expression has been demonstrated in various tissues by RT-PCR, antibody-based detection by Western blot or immunofluorescence has been unsuccessful, suggesting that *in vivo* protein levels are tightly regulated. Our findings suggest that a switch between ubiquitylation and SUMOylation affects Glis2/NPHP7 protein levels and function.

## Conclusions

The Glis2/NPHP7 transcriptional repressor, mutated in type 7 nephronophthisis, is modified by SUMOylation that inhibits its ubiquitylation and degradation. Tight control of Glis2/NPHP7 protein levels appears to be required to maintain epithelial cell homeostasis, explaining why this protein has so far evaded the detection of endogenous protein in native tissue.

## Supporting Information

S1 Fig Glis2 directly interacts with PIAS4. (PDF)
