# Supplementary material for: SUMOylation Blocks the Ubiquitin-Mediated Degradation of the Nephronophthisis Gene Product Glis2/NPHP7
Source: PLoS One. 2015 Jun 17;10(6):e0130275. doi: 10.1371/journal.pone.0130275 (PMC4471195; doi:10.1371/journal.pone.0130275)
Supplement: S1 Fig — (PDF) [file pone.0130275.s001.pdf]

# Supplementary Information

## Supplementary Figure Legends

**Figure S1. Glis2 directly interacts with PIAS4.** (A) F.Glis2, V5.PIAS4 and V5.CD2AP were expressed using cell free wheat germ extracts. Immuno-precipitation of V5.PIAS4 but not V5.CD2AP immobilized F.Glis2 (B) SUMO-3 fusion failed to inhibit the ubiquitylation of Glis2. HEK 293T cells were transfected with the plasmids as indicated. After 24 hours of transfection, F.Glis2 and F.SUMO-3.Glis2 were precipitated using FLAG-M2 beads; Glis2 ubiquitin species were detected in F.Glis2 as well as in F.SUMO-3.Glis2 immunoprecipitates (C) No change in protein stability was observed for the SUMO-3.Glis2 fusion protein. HEK 293T cells transfected with V5.Glis2 and V5.SUMO-3.Glis2 were treated with cycloheximide as indicated. (D) The graph demonstrates the fraction of Glis2 and SUMO-3.Glis2, remaining after 0 to 10 hours of cycloheximide treatment.

# Figure. S1

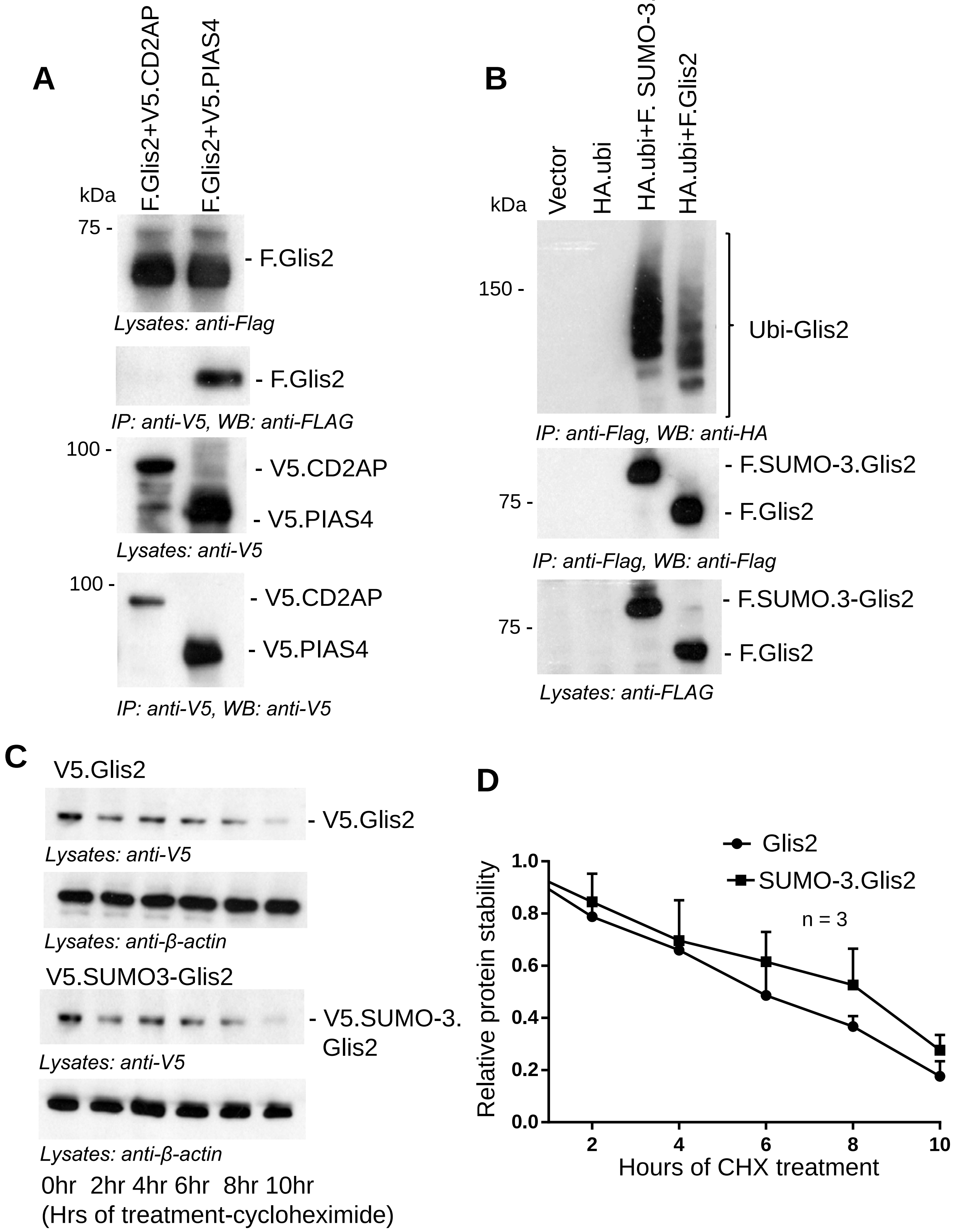
A
kDa
F.Glis2+V5.CD2AP
F.Glis2+V5.PIAS4
75 -
- F.Glis2
Lysates: anti-Flag
- F.Glis2
IP: anti-V5, WB: anti-FLAG
100 -
- V5.CD2AP
- V5.PIAS4
Lysates: anti-V5
100 -
- V5.CD2AP
- V5.PIAS4
IP: anti-V5, WB: anti-V5
B
kDa
Vector
HA.ubi
HA.ubi+F. SUMO-3.Glis2
HA.ubi+F.Glis2
150 -
Ubi-Glis2
IP: anti-Flag, WB: anti-HA
- F.SUMO-3.Glis2
75 -
- F.Glis2
IP: anti-Flag, WB: anti-Flag
- F.SUMO.3-Glis2
75 -
- F.Glis2
Lysates: anti-FLAG
C
V5.Glis2
- V5.Glis2
Lysates: anti-V5
Lysates: anti-β-actin
V5.SUMO3-Glis2
- V5.SUMO-3.
Glis2
Lysates: anti-V5
Lysates: anti-β-actin
0hr 2hr 4hr 6hr 8hr 10hr
(Hrs of treatment-cycloheximide)
D
Glis2
SUMO-3.Glis2
n = 3
Relative protein stability
1.0
0.8
0.6
0.4
0.2
0.0
2
4
6
8
10
Hours of CHX treatment
